# Supplementary material for: Tejas functions as a core component in nuage assembly and precursor processing in Drosophila piRNA biogenesis
Source: J Cell Biol. 2023 Aug 9;222(10):e202303125. doi: 10.1083/jcb.202303125 (PMC10412688; doi:10.1083/jcb.202303125)
Supplement: Table S1 — lists Drosophila genotypes used in this study. [file JCB_202303125_TableS1.docx]

**Supplementary Table 1. *Drosophila* genotypes used in this study.**

Fig.1A:

*w[-]; tej[EGFP.KI]*/ *CyO; spn-E[mKate2.KI]*/ *TM3, Sb*

*w[-]; tej[EGFP.KI], vas[mCherry.HA.KI]*/ *CyO*

Fig.1B, S1B:

*w[-]; tej^48-5^, vas[EGFP.KI]*/ *CyO*

*w[-]; tej^48-5^, aub[EGFP.KI]*/ *CyO*

*w[-]; tej^48-5^*/ *CyO*; *spn-E[mKate2.KI]*/ *TM3, Sb*

*w[-]; tej^48-5^*/ *CyO*; *ago3[mKate2.KI]*/ *TM3, Sb*

Fig.1C, S1C:

*w[-]; tej^48-5^, vas[EGFP.KI]*/ *CyO*

*w[-]; tej^48-5^*/ *CyO* ; *spn-E[mKate2.KI]*/ *TM3, Sb*

Fig.1D:

*w[-]; vas[EGFP.KI]*/ *CyO*

*w[-]; tej[EGFP.KI]*/ *CyO*

*w[-]; spn-E[mKate2.KI]*/ *TM3, Sb*

Fig.S1A:

*w[-]; vas[EGFP.KI]*/ *CyO*

*w[-]; tej[EGFP.KI]*/ *CyO*

*w[-]; ago3[mKate2.KI]*/ *TM3, Sb*

Fig.S1D:

*w[-]; vas^PH165^*/ *CyO; spn-E[mKate2.KI]*/ *TM3, Sb*

*w[-]; vas[mCherry.HA.KI]*/ *CyO; spn-E^616^*/ *TM3, Sb*

*w[-]; Df(2L)BSC299*/ *CyO*

*w[-]; Df(3R)Exel8162*/ *TM3, Sb*

Fig.2E:

*w[-]; spn-E[mKate2.KI]*/ *TM3, Sb*

*w[-]; traffic jam*-Gal4/ *CyO; nos*-Gal4 VP16, *Df(3R)Exel8162*/ *TM3, Sb*

*w[-];* UASp-GFP-Spn-E^wt^/ *CyO*; *spn-E^616^*/ *TM3, Sb*

*w[-];* UASp-GFP-Spn-E^ΔNLS^/ *CyO; spn-E^616^*/ *TM3, Sb*

Fig.3A, 3B, S4A-B:

*w[-]; tej^48-5^*/ *CyO*

*w[-]; tej^48-5^*/ *CyO* ; *ago3[mKate2.KI]*/ *TM3, Sb*

Fig.3C-E:

*y[-]w[-]*

*w[-]; tej^48-5^*/ *CyO*

*w[-]; vas^PH165^*/ *CyO*

*w[-]; spn-E^616^*/ *TM3, Sb*

*w[-]; krimp^f06583^*/ *CyO*

*w[-]; nxf3^Δ^* /*TM6C, Sb1*

*w[-]; Df(2L)BSC299*/ *CyO*

*w[-]; Df(3R)Exel8162*/ *TM3, Sb*

Fig.4B-F, S5A-C:

*w[-]; tej^48-5^, NGT40-*Gal4/ *CyO; nos-*Gal4 VP16

*w[-]; tej^48-5^, NGT40-*Gal4/ *CyO; nos-*Gal4 VP16*, spn-E[mKate2.KI]*

*w[-]; tej^48-5^, NGT40-*Gal4/ *CyO, vas[EGFP.KI]; nos-*Gal4 VP16

*w[-]; tej^48-5^*/ *CyO*; UASp-miniTurbo-GFP-Tej-FL

*w[-]; tej^48-5^*/ CyO; UASp-miniTurbo-GFP-Tej-ΔLotus

*w[-]; tej^48-5^*/ *CyO*; UASp-miniTurbo-GFP-Tej-ΔeSRS

*w[-]; tej^48-5^*/ *CyO*; UASp-miniTurbo-GFP-Tej-ΔeTudor

*w[-]; tej^48-5^*/ *CyO*; UASp-GFP-Tej-ΔSRS

Fig.5E:

*w[-]; tej^48-5^, NGT40-*Gal4/ *CyO; nos-*Gal4 VP16

*w[-]; tej^48-5^*/ *CyO*; UASp-miniTurbo-GFP-Tej-FL

*w[-]; tej^48-5^*/ *CyO*; UASp-GFP-Tej-ΔIDR

Fig.5G:

*w[-]; tej^48-5^, NGT40-*Gal4/ *CyO, vas[EGFP.KI]; nos-*Gal4 VP16

*w[-]; tej^48-5^*/ *CyO*; UASp-miniTurbo-GFP-Tej-FL

*w[-]; tej^48-5^*/ *CyO*; UASp-GFP-Tej-ΔIDR

Fig.S6C:

*w[-]; tej^48-5^, NGT40-*Gal4/ *CyO; nos-*Gal4 VP16

*w[-]; tej^48-5^*/ *CyO*; UASp-miniTurbo-GFP-Tej-FL

*w[-]; tej^48-5^*/ *CyO*; UASp-GFP-Tej-ΔIDR
