# Supplementary material for: Tejas functions as a core component in nuage assembly and precursor processing in Drosophila piRNA biogenesis
Source: J Cell Biol. 2023 Aug 9;222(10):e202303125. doi: 10.1083/jcb.202303125 (PMC10412688; doi:10.1083/jcb.202303125)
Supplement: Table S2 — lists antibodies used in this study. [file JCB_202303125_TableS2.docx]

**Supplementary Table 2. List of antibodies used in this study.**

| Antibody Name | Source |
| --- | --- |
| Anti-Tej Rat polyclonal antibody | Current study |
| Anti-Vas Guinea pig polyclonal antibody | (Patil and Kai, 2010) |
| Anti-Spn-E Rat polyclonal antibody | (Patil and Kai, 2010) |
| Anti-Ago3 Rat polyclonal antibody | Current study |
| Anti-Piwi mouse monoclonal antibody | (Saito et al., 2006) |
| Anti-Ago2 Guinea pig polyclonal antibody | (Iki et al., 2020) |
| Anti-Myc Mouse monoclonal antibody | Wako, Cat.# 017-21871 |
| Anti-eGFP Mouse monoclonal antibody | Invitrogen, Cat.# A-11120 |
| Anti-mKate2 Mouse monoclonal antibody | Evrogen, Cat.# AB231 |
| Anti-HetA Rabbit polyclonal antibody | Current study |
| Anti-Fibrillarin Rabbit polyclonal antibody | Abcam, Cat.# ab5821 |
| Anti-Aub Guinea pig polyclonal antibody | (Lim et al., 2022) |
| HRP-conjugated goat anti-guinea pig | Dako, Cat.# P0141 |
| HRP-conjugated goat anti-rat | Dako, Cat.# P0450 |
| HRP-conjugated goat anti-mouse | BioRad, Cat.# 1706516 |
| HRP-conjugated goat anti-rabbit | BioRad, Cat.# 1706515 |
