# Supplementary material for: Tejas functions as a core component in nuage assembly and precursor processing in Drosophila piRNA biogenesis
Source: J Cell Biol. 2023 Aug 9;222(10):e202303125. doi: 10.1083/jcb.202303125 (PMC10412688; doi:10.1083/jcb.202303125)
Supplement: Table S3 — lists primers used for qRT-PCR in this study. [file JCB_202303125_TableS3.docx]

**Supplementary Table 3. List of primers used for qRT-PCR in this study.**

| Primer label | Primer sequence | Source |
| --- | --- | --- |
| Rp49_Fw | ATGACCATCCGCCCAGCATAC | Current study |
| Rp49_Rv | CTGCATGAGCAGGACCTCCAG | Current study |
| Tubulin_Fw | GGTAACCGTCGAAATCAGTGTT | Current study |
| Tubulin_Rv | TGGCTTTTCTGCTATACGTGTC | Current study |
| 38C #1_Fw | GAGACTTGCCGTTCCTTAG | Current study |
| 38C #1_Rv | CCATCTGGAATGCAAACG | Current study |
| 38C #2_Fw | TCCGTGACGGTTTAGCCCA | Current study |
| 38C #2_Rv | AGGTTTCAAACCTTCCAG | Current study |
| 42AB #1_Fw | CGTCCCAGCCTACCTAGTCA | (ElMaghraby et al., 2019) |
| 42AB #1_Rv | ACTTCCCGGTGAAGACTCCT | (ElMaghraby et al., 2019) |
| 42AB #2_Fw | CGCTGTTGAAAGCAAATTGA | (ElMaghraby et al., 2019) |
| 42AB #2_Rv | GAGACCTTCGCTCCAGTGTC | (ElMaghraby et al., 2019) |
| Flam #1_Fw | ACGCTCAGGAAGGGATTTCA | Current study |
| Flam #1_Rv | AAACATGTCGTCTATCCATC | Current study |
| Flam #2_Fw | TCTCGGATAGAACTCTTCCC | Current study |
| Flam #2_Rv | TTGAACCTGTAGGCTAGGTA | Current study |
| HeT-A_Fw | ACAGATGCCAAGGCTTCAGG | (Piñeyro et al., 2011) |
| HeT-A_Rv | GCCAGCGCATTTCATGC | (Piñeyro et al., 2011) |
| TART_Fw | TTCTATCAACAGGCTGTCCACAGGTT | (Savitsky et al., 2006) |
| TART_Rv | CCTTCGTAGTCGGGTAGGATTATTCGT | (Savitsky et al., 2006) |
| TAHRE_Fw | CTGTTGCACAAAGCCAAGAA | (Chen et al., 2016) |
| TAHRE_Rv | GTTGGTAATGTTCGCGTCCT | (Chen et al., 2016) |
| I-element | TGAAATACGGCATACTGCCCCCA | (Klenov et al., 2011) |
| I-element | GCTGATAGGGAGTCGGAGCAGATA | (Klenov et al., 2011) |
