# Supplementary material for: Tejas functions as a core component in nuage assembly and precursor processing in Drosophila piRNA biogenesis
Source: J Cell Biol. 2023 Aug 9;222(10):e202303125. doi: 10.1083/jcb.202303125 (PMC10412688; doi:10.1083/jcb.202303125)

SourceDataF1

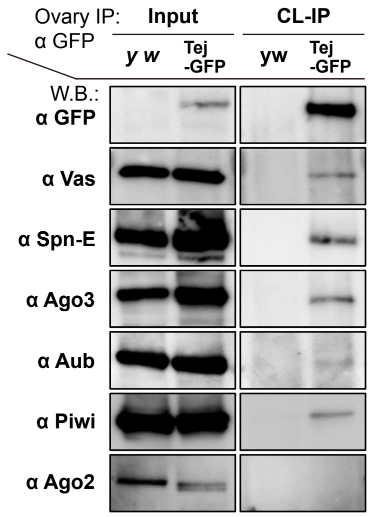

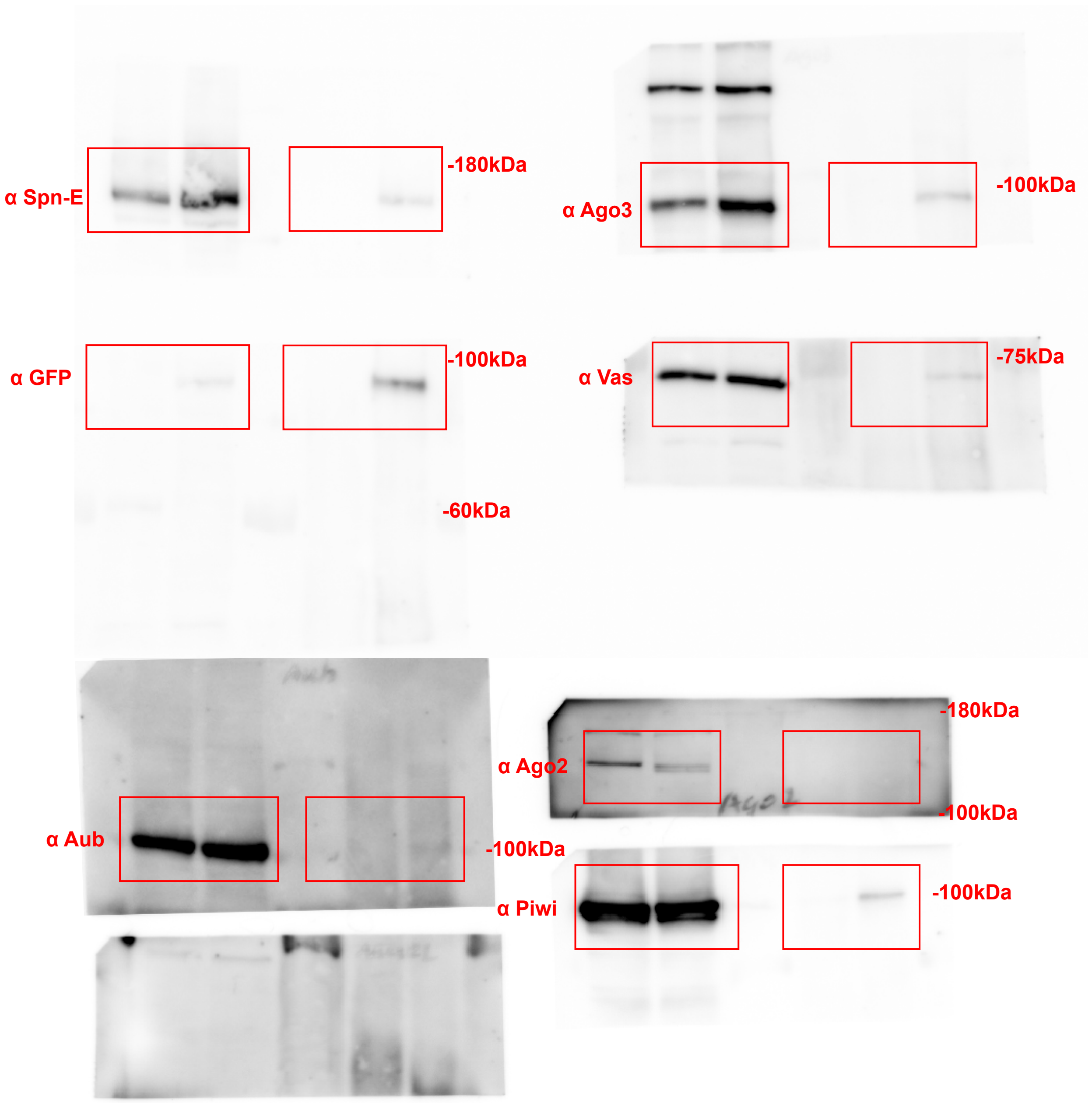

SourceDataF1

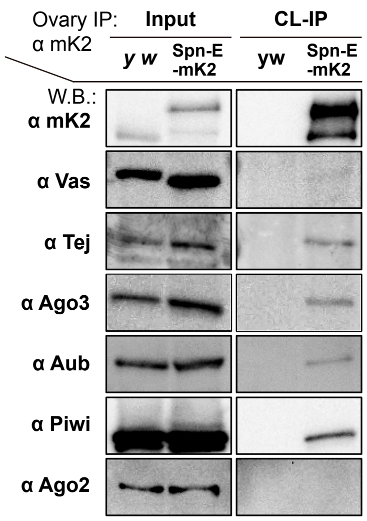

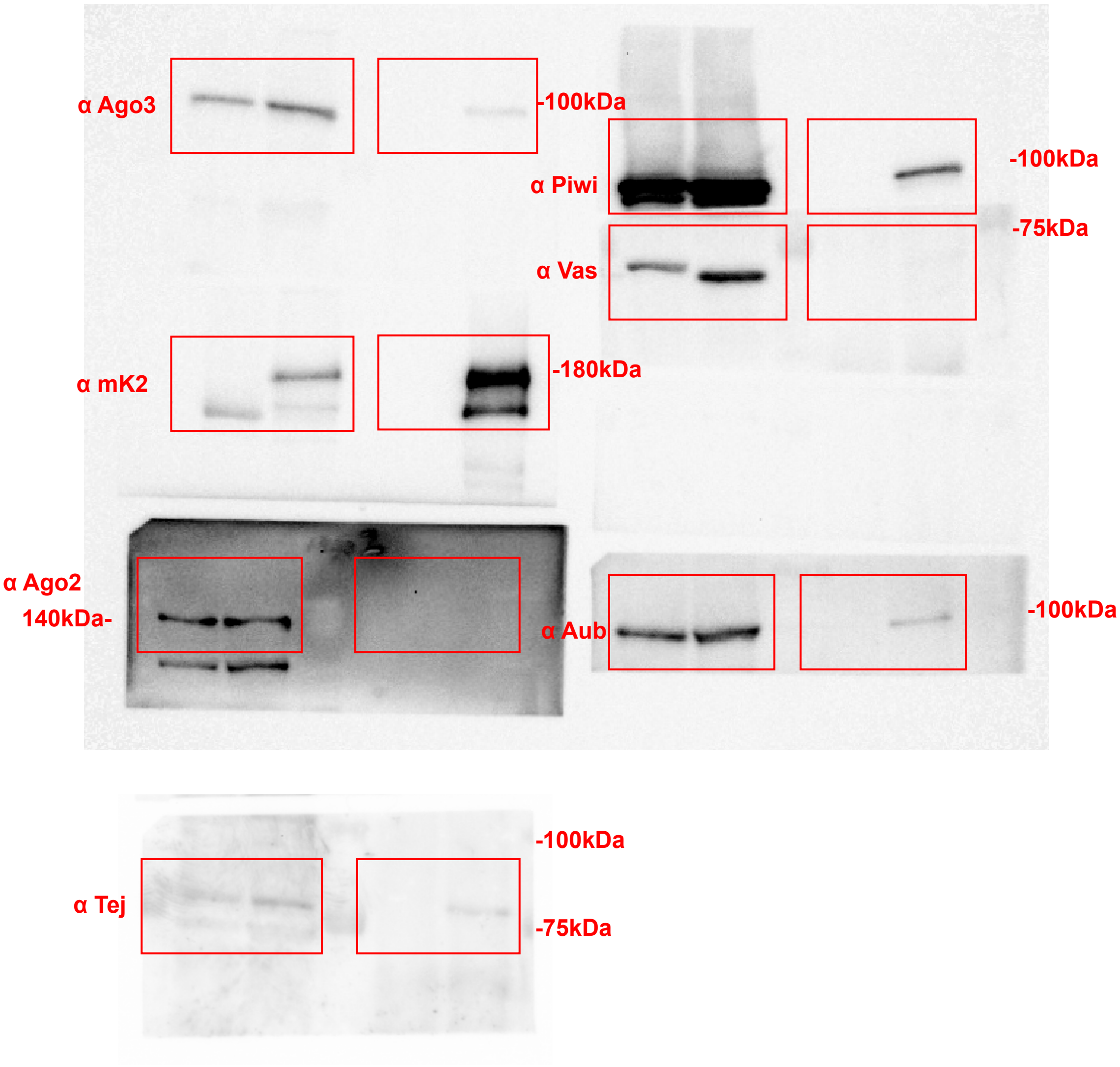

SourceDataF1

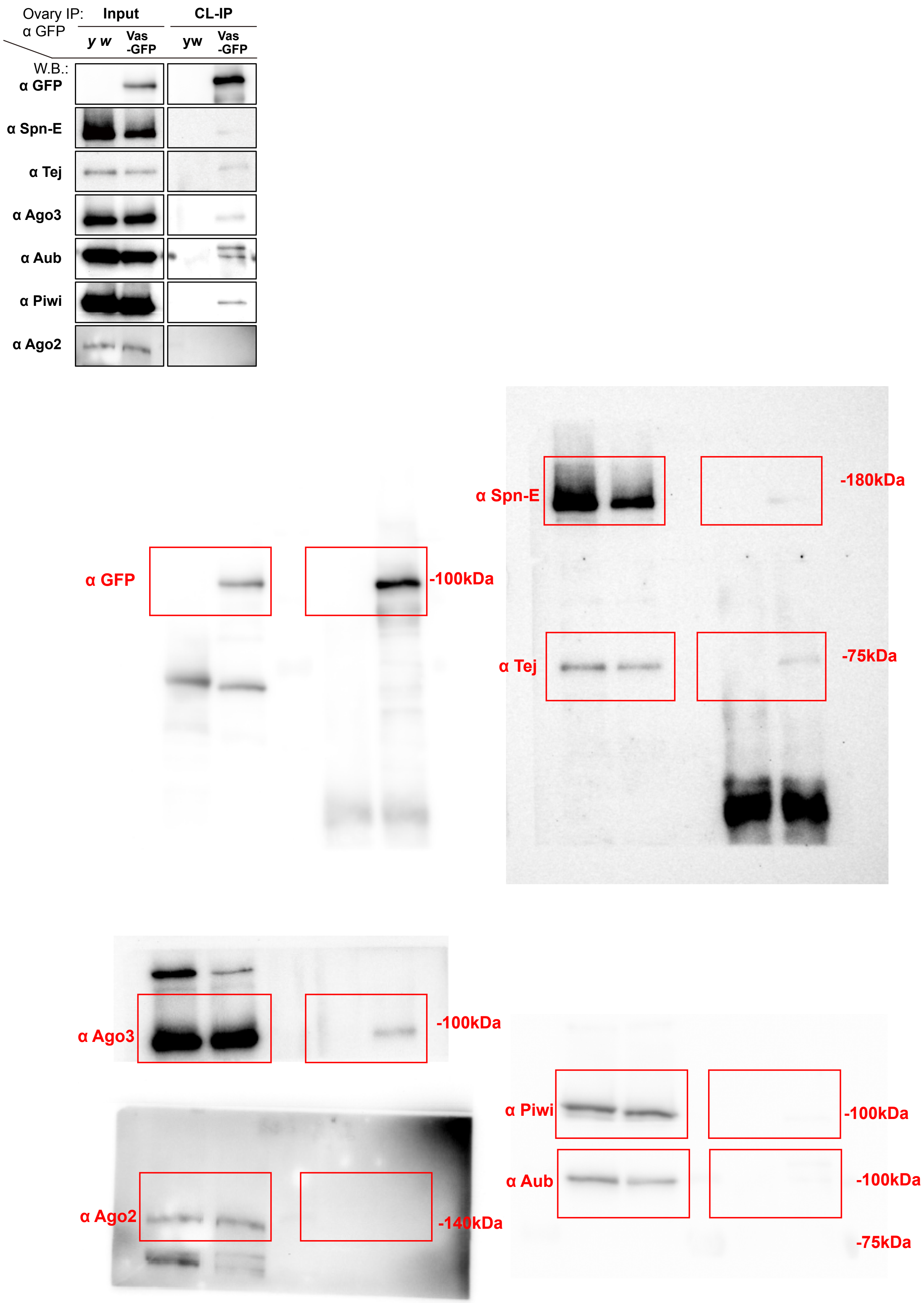

Supplement: SourceData F1 — is the source file for Fig. 1. [file JCB_202303125_SourceDataF1.pdf]
