# Supplementary figures and images for: Tejas functions as a core component in nuage assembly and precursor processing in Drosophila piRNA biogenesis
Source: J Cell Biol. 2023 Aug 9;222(10):e202303125. doi: 10.1083/jcb.202303125 (PMC10412688; doi:10.1083/jcb.202303125)

# SourceDataSF4

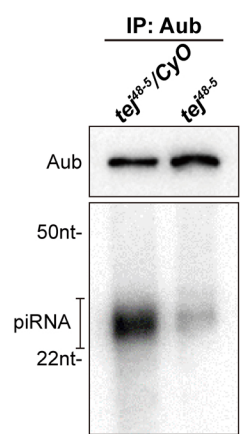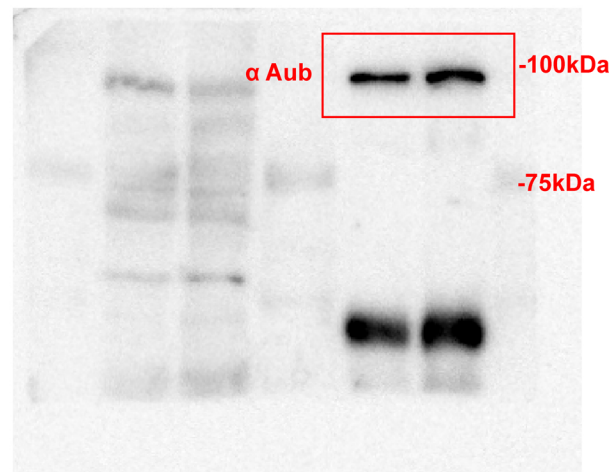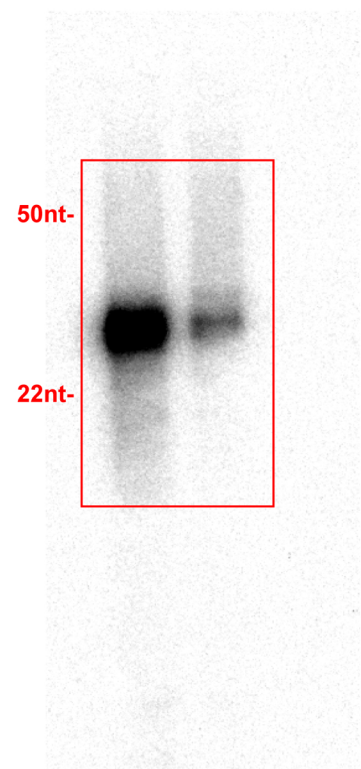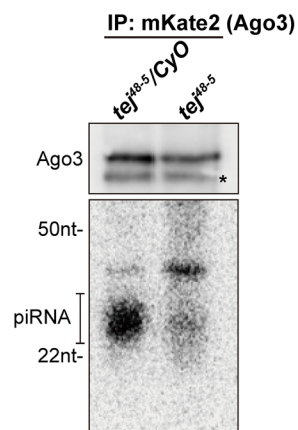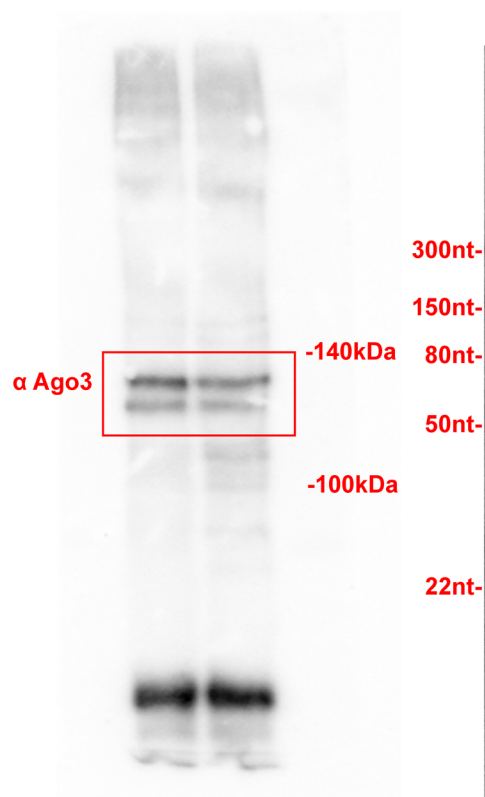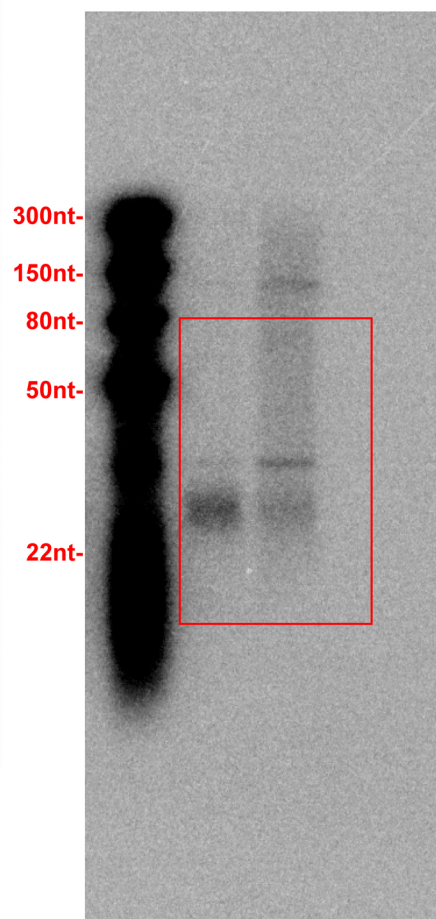

Supplement: SourceData FS4 — is the source file for Fig. S4. [file JCB_202303125_SourceDataFS4.pdf]

# SourceDataSF5

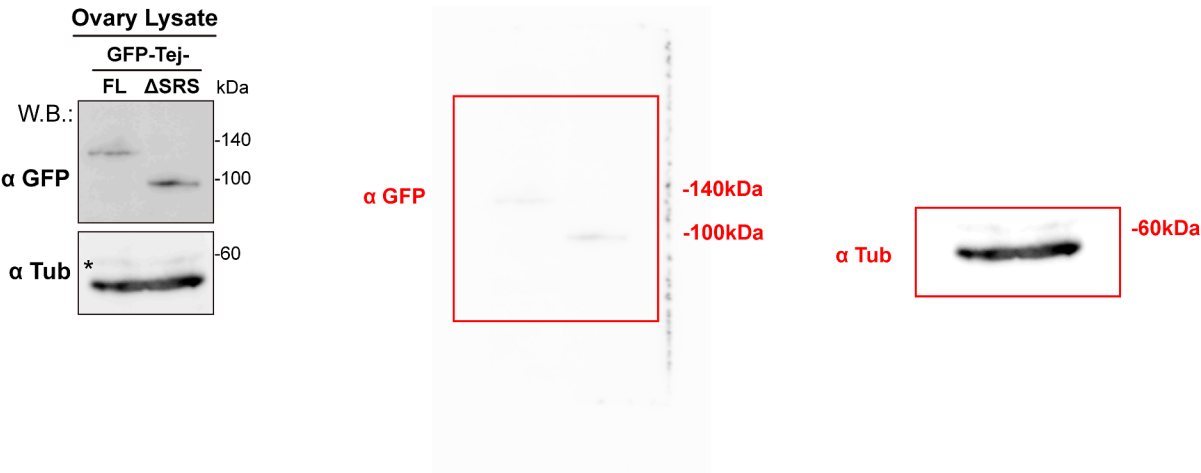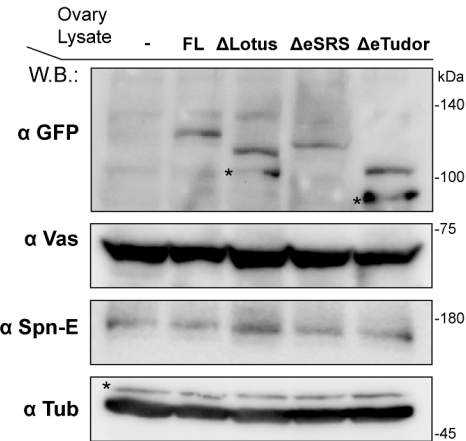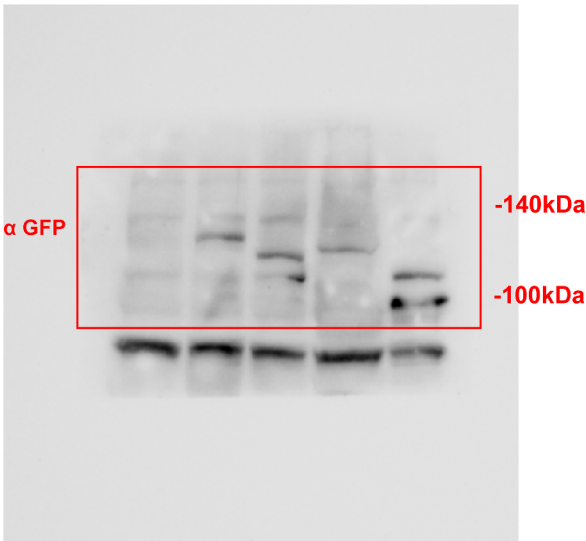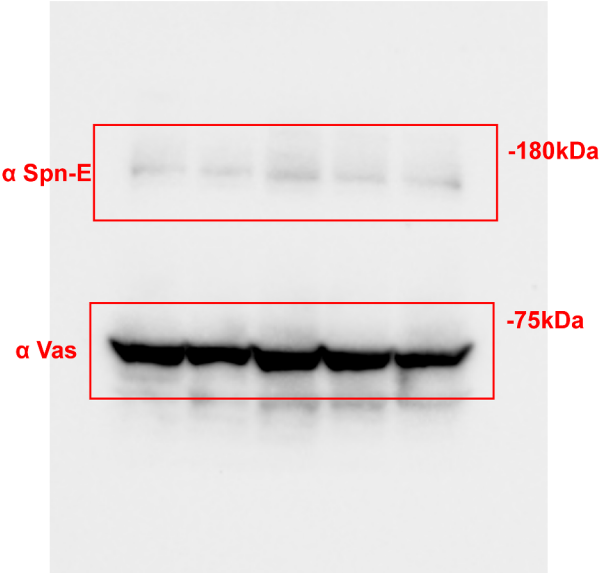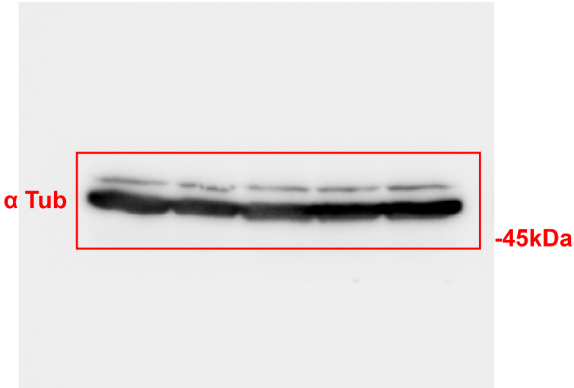

# SourceDataSF5

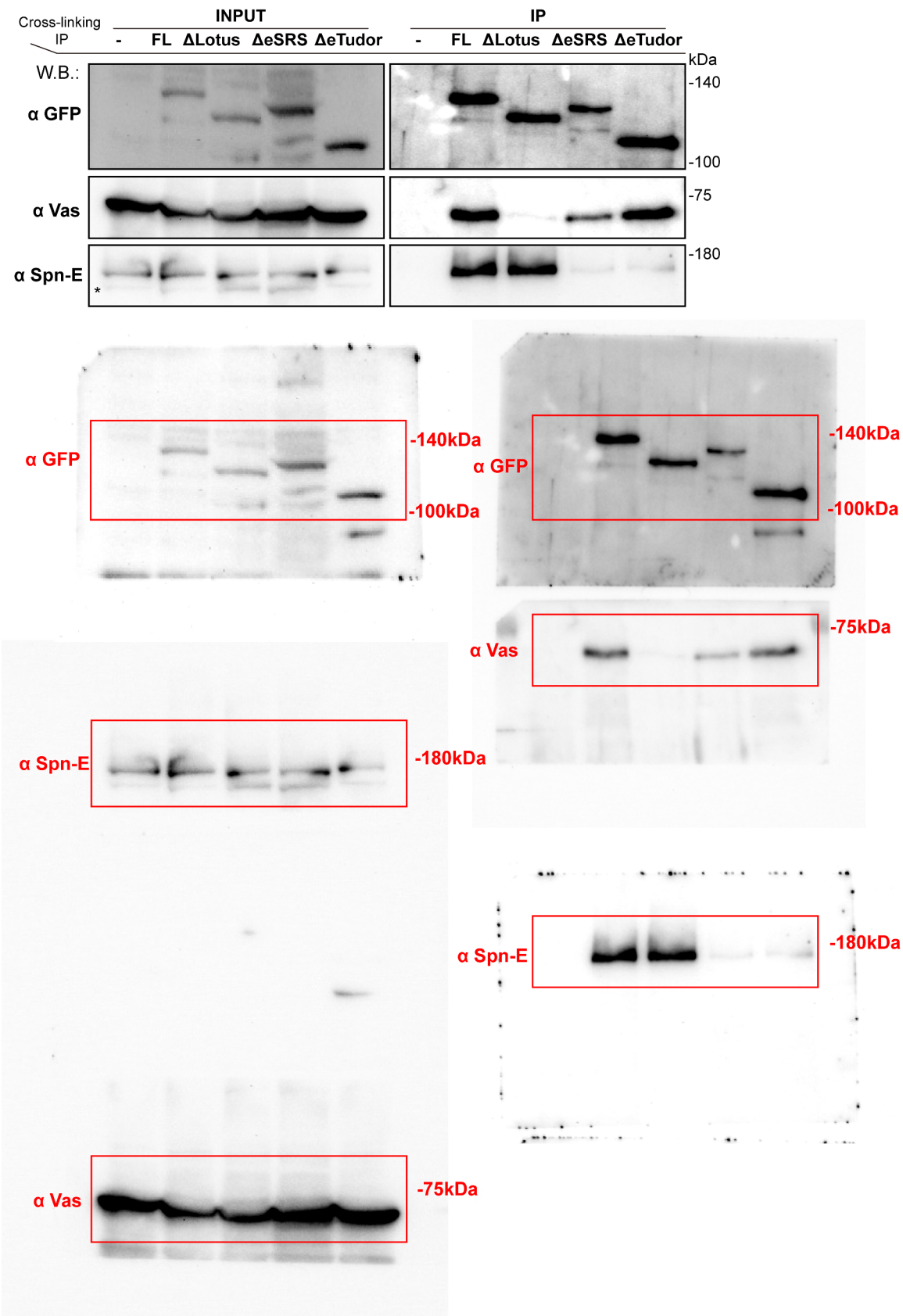

Supplement: SourceData FS5 — is the source file for Fig. S5. [file JCB_202303125_SourceDataFS5.pdf]

# SourceDataSF6

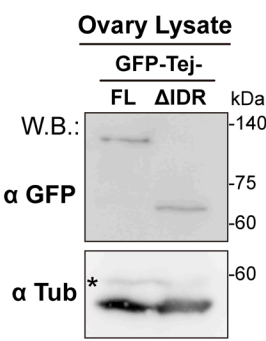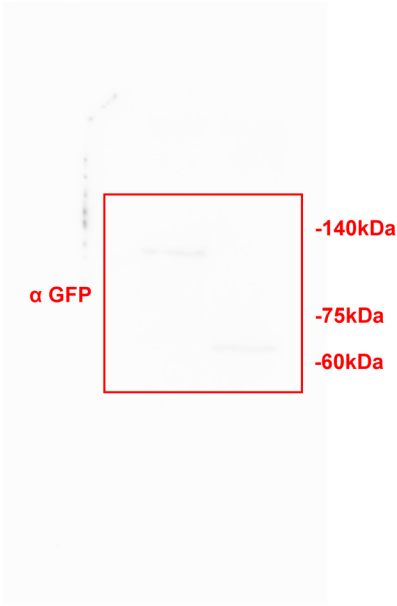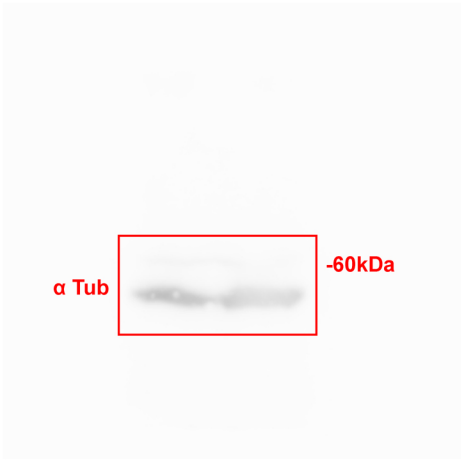

Supplement: SourceData FS6 — is the source file for Fig. S6. [file JCB_202303125_SourceDataFS6.pdf]
